# Supplementary material for: The folklore of the “Swift” effect – lessons for medical research and clinical practice
Source: PLoS One. 2025 Sep 19;20(9):e0315560. doi: 10.1371/journal.pone.0315560 (PMC12448345; doi:10.1371/journal.pone.0315560)
Supplement: S3 File — This includes two supplemental tables: Table S1. Sensitivity analysis for models examining the effects of Swifts presence or absence on Travis Kelce’s performance. Table S2. Sensitivity analysis for models examining the effects of Swifts presence or absence on Kansas City Chief’s win probability. (DOCX) [file pone.0315560.s003.docx]

**Table S1. Sensitivity analysis for models examining the effects of Swifts presence or absence on Travis Kelce’s performance.**

| **Matching Algorithm** | **Model** | **Intercept**  **(95% confidence interval)**  **[p-value]** | **Eras**  **(95% confidence interval)**  **[p-value]** | **Pre-Game Elo**  **(95% confidence interval)**  **[p-value]** |
| --- | --- | --- | --- | --- |
| 1:1 non-replacement | Swift Present | -851.0  (-1265.8, -436.1)  [<0.001] | -0.1  (-35.4, 35.2)  [0.995] | 0.561  (0.309, 0.814)  [<0.001] |
| 1:1 with replacement | Swift Present | -542.4  (1035.3, -49.4)  [0.034] | 3.2  (-35.9, 42.3)  [0.860] | 0.373  (0.073, 0.673)  [0.019] |
| 2:1 non-replacement | Swift Present | -596.6  (-1072.5, -120.8)  [0.016] | -3.3  (-25.2, 18.7)  [0.760] | 0.410  (0.123, 0.700)  [0.007] |
| 2:1 with replacement | Swift Present | -31.1  (-587.8, 525.7)  [0.909] | -1.7  (-28.2, 24.8)  [0.897] | 0.068  (-0.268, 0.404)  [0.680] |
| 3:1 non-replacement | Swift Present | -363.0  (-1016.5, 290.5)  [0.268] | -2.5  (-23.5, 18.5)  [0.812] | 0.269  (-0.125, 0.663)  [0.175] |
| 3:1 with replacement | Swift Present | -183.7  (-641.1, 273.7)  [0.421] | -2.0  (-26.0, 22.0)  [0.867] | 0.160  (-0.115, 0.346)  [0.247] |
| 5:1 non-replacement | Swift Present | -131.1  (-450.1, 187.8)  [0.414] | 7.1  (-12.7, 26.9)  [0.476] | 0.123  (0.12, 0.32)  [0.204] |
| 5:1 with replacement | Swift Present | -135.0  (-424.6, 154.7)  [0.355] | 2.6  (-19.8, 25.0)  [0.818] | 0.128  (-0.047, 0.303)  [0.148] |
| 8:1 non-replacement | Swift Present | -249.4  (-495.6, -3.111)  [0.047] | 5.7  (-14.7, 26.1)  [0.579] | 0.194  (0.045, 0.345)  [0.012] |
| 8:1 with replacement | Swift Present | -0.855  (-250,5, 248.8)  [0.995] | 5.0  (-15.7, 25.7)  [0.635] | 0.046  (-0.105, 0.196)  [0.548] |
|  |  |  |  |  |
| 1:1 non-replacement | Swift Absent | -63.2  (-1952.91, 1826.6)  [0.935] | -11.9  (-52.3, 28.6)  [0.462] | 0.075  (-1.14, 1.29)  [0.873] |
| 1:1 with replacement | Swift Absent | 1022.8  (-762.3, 2807.9)  [0.201] | -45.3  (-92.9, 2.3)  [0.057] | -0.553  (-1.702, 0.596)  [0.253] |
| 2:1 non-replacement | Swift Absent | 33.83  (-1441.3, 1508.9)  [0.960] | -42.5  (-59.6, -25.3)  [<0.001] | 0.022  (-0.858, 0.901)  [0.957] |
| 2:1 with replacement | Swift Absent | 329.9  (-1339.2, 1999.0)  [0.669] | -29.9  (-69.0, 9.3)  [0.120] | -0.151  (-1.146, 0.844)  [0.742] |
| 3:1 non-replacement | Swift Absent | 285.6  (-1675.1, 2246.3)  [0.761] | -15.7  (-48.5, 17.0)  [0.324] | -0.130  (-1.299, 1.039)  [0.816] |
| 3:1 with replacement | Swift Absent | 276.5  (993.3, 1546.3)  [0.651] | -25.0  (-58.5, 8.5)  [0.133] | -0.120  (-0.877, 0.637)  [0.741] |
| 5:1 non-replacement | Swift Absent | 116.2  (-1201.4, 1433.8)  [0.858] | -28.6  (-69.4, 12.3)  [0.163] | -0.023  (-0.81, 0.76)  [0.954] |
| 5:1 with replacement | Swift Absent | 66.6  (-1348.5, 1481.7)  [0.924] | -25.0  (-53.3, 3.3)  [0.081] | 0.003  (-0.841, 0.847)  [0.995] |
| 8:1 non-replacement | Swift Absent | 20.7  (-418.1, 459.5)  [0.925] | -34.1  (-65.0, -3.2)  [0.031] | 0.038  (-0.225, 0.301)  [0.773] |
| 8:1 with replacement | Swift Absent | -206.5  (-1175.1, 762.2)  [0.670] | -25.8  (-54.1, 2.4)  [0.072] | 0.169  (-0.408, 0.747)  [0.558] |

**Table S2. Sensitivity analysis for models examining the effects of Swifts presence or absence on Kansas City Chief’s win probability.** Values are presented as odds ratios, whereby 1.0 represents an equal chance of win or loss, and values above 1.0 represent an increased win probability when Taylor Swift was present.

| Matching Algorithm | Chiefs Win Probability  (95% confidence interval)  [p-value] |
| --- | --- |
| 1:1 non-replacement | 0.97 (0.31, 9.49) (p=0.532) |
| 1:1 with replacement | 1.05 (0.22, 5.13) (p=0.949) |
| 2:1 non-replacement | 1.09 (0.22, 5.40) [p=0.916] |
| 2:1 with replacement | 0.98 (0.22, 4.30) [p=0.977] |
| 3:1 non-replacement | 1.04 (0.25, 4.29) [p=0.955] |
| 3:1 with replacement | 1.04 (0.25, 4.29) [p=0.955] |
| 5:1 non-replacement | 1.33 (0.33, 5.34) [p=0.692] |
| 5:1 with replacement | 1.10 (0.28, 4.30) [p=0.895] |
| 8:1 non-replacement | 1.27 (0.31, 5.17) [p=0.742] |
| 8:1 with replacement | 1.48 (0.38, 5.82) [p=0.575] |
